# Within and combined season prediction models for perennial ryegrass biomass yield using ground- and air-based sensor data

**DOI:** 10.3389/fpls.2022.950720

**Published:** 2022-08-08

**Authors:** Phat T. Nguyen, Fan Shi, Junping Wang, Pieter E. Badenhorst, German C. Spangenberg, Kevin F. Smith, Hans D. Daetwyler

**Affiliations:** ^1^School of Applied System Biology, La Trobe University, Bundoora, VIC, Australia; ^2^Agriculture Victoria, AgriBio, Centre for AgriBioscience, Bundoora, VIC, Australia; ^3^Agriculture Victoria, Hamilton Centre, Hamilton, VIC, Australia; ^4^Faculty of Veterinary and Agricultural Sciences, School of Agriculture and Food, The University of Melbourne, Melbourne, VIC, Australia

**Keywords:** perennial ryegrass, cross-season yield, high-throughput phenotyping, sensor, prediction model, unmanned vehicle, uncrewed

## Abstract

Across-season biomass assessment is crucial in the cultivar selection process to accurately evaluate the yield performance of lines under different growing conditions. However, it has been difficult to have an accurate, reliable, and repeated fresh biomass (FM) estimation of large populations of plants in the field without destructive harvesting, which incurs significant labor and operation costs. Sensor-based phenotyping platforms have advanced in the data collection of structural and vegetative information of plants, but the developed prediction models are still limited by low correlations at different growth stages and seasons. In this study, our objective was to develop and validate the global prediction models for across-season harvested fresh biomass (FM) yield based on the ground- and air-based sensor data including ground-based LiDAR, ground-based ultrasonic, and air-based multispectral camera to extract LiDAR plant volume (LV), LiDAR point density (LV_Den), height, and Normalized Difference Vegetative Index (NDVI). The study was conducted in a row-plot field trial with 480 rows (3 rows in a plot per cultivar) throughout the whole 2020 growing season up to the reproductive stage. We evaluated the performance of each plant parameter, their relationship, and the best subset prediction models using statistical stepwise selection at the row and plot levels through the seasonal and combined seasonal datasets. The best performing model: FM~LV∗LV_Den∗NDVI had a determination of coefficient *R*^2^ of at least 0.9 in vegetative stages and 0.8 in the reproductive stage. Similar results can be achieved in a simpler model with just two LiDAR variables— FM~LV∗LV_Den . In addition, LV and LV_Den showed a robust correlation with FM on their own over seasons and growth stages, while NDVI only performed well in some seasons. The simpler model based on only LiDAR data can be widely applied over season without the need of additional sensor data and may thus make the in-field across-season biomass assessment more feasible and practical for fast and cost-effective development of higher biomass yield cultivars.

## Introduction

Climate change poses a sustained challenge to have better forage cultivars with a higher yield and resilience to secure feed availability and grazing profit, in which perennial ryegrass is the most important forage species in temperate regions (Wilkins, 1991; Cunningham et al., 1994). The current selection of cultivars relies on the evaluation of biomass production in the field. Across-season yield assessment is essential to evaluate the robust yield performance under different growing conditions, which results in a long breeding cycle of up to 15 years (Hayes et al., 2013; McDonagh et al., 2016). New modern breeding methods such as genomic selection (GS) can shorten the breeding cycle (Lin et al., 2016), but it requires a large training population planted in different trial configurations (e.g., spaced plants, row-based plots, or sward-based plots) at early cultivar development stages to verify the genetic variation between/within experimental cultivars (Lin et al., 2014; Annicchiarico et al., 2015). Accurate biomass estimation is thus important to measure a diverse biomass performance and understand how genotypes respond to the growing condition. However, while destructive methods are the most accurate to estimate the actual biomass (Catchpole and Wheeler, 1992), they are laborious, time-consuming, and costly, limiting the size of field trials. Consequently, the inability of assessing accurate biomass estimation from large populations in the field has limited the rapid development of new cultivars using genomic selection breeding methods (Pembleton et al., 2018; Ghamkhar et al., 2019).

Remote sensing approaches are non-destructive, high-throughput, and efficient methods able to accurately screen large numbers of plants at different levels of spatial resolution (Roy and Ravan, 1996; Kumar et al., 2015; Lu et al., 2016). To support large-scale measurement, various high-throughput phenotyping platforms have integrated remote sensing to increase data acquisition capabilities from the ground, air, and satellites, reducing operation cost and time capture as well as providing repeated non-destructive measurement in the field. Despite the effective and unlimited access to large areas across the globe, satellite-based platforms are limited on spatial and temporal resolution. Ground- and air-based platforms have advantages for proximal assessment, which is necessary for the experimental breeding trials often containing rows and small plots. Therefore, uncrewed ground (UGV) and aerial vehicles (UAV) have been developed and validated for a range of important agronomic traits in agriculture in crops and forage species (Dungey et al., 2018; Gebremedhin et al., 2019b; Zhao et al., 2019). However, currently crewed ground-based platforms (tractors and side-by-side vehicles) remain the primary platform for pasture measurements. The type of phenotyping platform used depends on the trial configuration and size as well as the field of application.

A variety of remote sensors (proximal, optical, multispectral, and hyperspectral sensors) have been deployed to measure plant canopy structure (i.e., plant height, diameter, density, etc.) and vegetation indexes (VIs) to determine their empirical relationship with desirable traits. These digital parameters have been demonstrated to be useful in the estimation of agronomic traits in crop and forest species, including biomass. For grass species, biomass estimates from canopy structure parameters or vegetative indexes are different to crops and vary widely over the growth stages/season and the level of spatial and temporal resolution due to their heterogeneous morphology and structure (Zhang et al., 2018). Previous studies have addressed this issue by combining/incorporating canopy structure parameters and vegetative indexes to create more robust biomass prediction models (Sbrissia et al., 2001; Li et al., 2015; Andersson et al., 2017; Gebremedhin et al., 2019a; Zhang et al., 2019). Most recent prediction models were developed using conventional regression methods, especially multiple linear regressions, to combine the plant height (PH), canopy height metrics (CHM), and point-derived plant density with NDVI and other VIs (Schaefer and Lamb, 2016; Andersson et al., 2017; Moeckel et al., 2017; Yue et al., 2018; Gebremedhin et al., 2019a). Better correlations were observed, and significant correlations were reported at the sward/paddock level, but they were still low at single plant and row-based levels (Gebremedhin et al., 2019a; Wang et al., 2019). In general, studies have proposed seasonal models and across-season prediction models have not been developed to date.

New robust parameters are desirable in biomass estimation of forage grasses as well as simplifying the prediction models. In recent years, plant volume has gained attention as a proxy of biomass, as plant response is strongly linked with its physical three-dimensional (3D) structural space (Omasa et al., 2007). Measuring plant volume can accurately estimate biomass production and could potentially replace a number of diverse parameters such as NDVI during the whole growing cycle. Good results of estimated biomass from plant volume were reported in maize (Han et al., 2019), wheat (Walter et al., 2019), grapevine (Keightley and Bawden, 2010), shrubs, and short herbaceous plants (Hirata et al., 2007). For perennial ryegrass, few studies using manually collected data have shown a strong correlation between plant volume and harvested biomass in controlled environments (Wang et al., 2020) and field conditions (Ghamkhar et al., 2019). Recently, we have developed and validated an uncrewed ground vehicle integrated with a LiDAR sensor to measure plant volume in row-plot field trials (Nguyen et al., 2021). Ghamkhar et al. (2019) pointed out that the biomass estimates using LiDAR plant volume showed a small variation across season and low correlation at the high-density biomass at the end of growing period. This could be explained by the lack of a density parameter in the context of the mass theory. The density of ryegrass is complex and driven by many physiological factors such as tiller number, leaf area, water content, and chlorophyll content. In the past, studies used the point-derived plant density from LiDAR data to compensate for the disadvantages of VIs, such as saturation at the high biomass and less accuracy due to soil effects at low biomass levels. However, the use of VIs, particularly NDVI, is widely used as it strongly correlates with living biomass during the vegetative stage.

The main objectives of this study were to (1) review and evaluate the empirical relationship between each digital parameter (PH, plant volume, plant density, and NDVI) and harvested fresh biomass (FM), (2) develop and evaluate a variety of models using LiDAR data with other plant phenomic parameters, and (3) select and validate the best model with statistical cross-validation methods to define its reliability and robustness for within and across-season ryegrass biomass estimation.

## Materials and methods

### Field experiment

**Figure 1:**
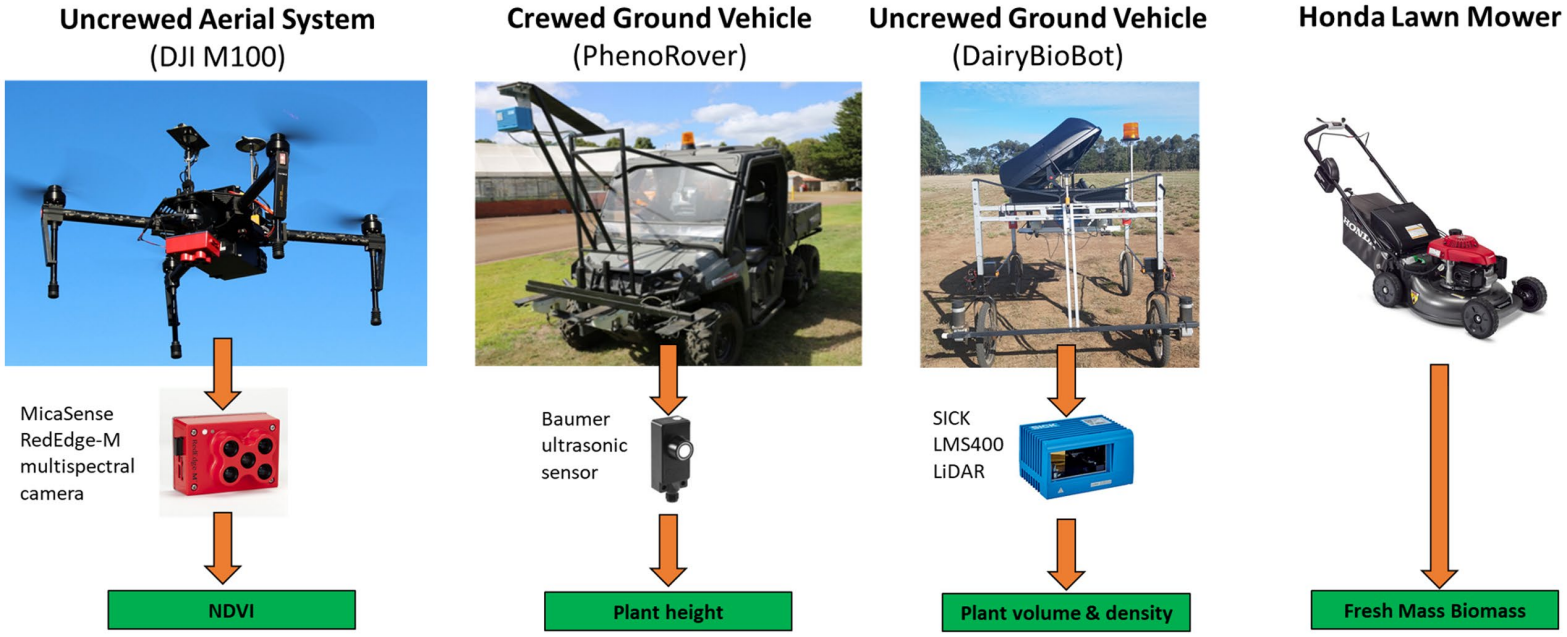
Aerial and ground-based high-throughput phenotyping platforms used in this study to estimate an empirical relationship with harvested fresh biomass from a manual lawn mower.

The experiment was conducted at Agriculture Victoria Research, Hamilton SmartFarm, Hamilton, Victoria, Australia (−37.8468, 142.0743). A perennial ryegrass breeding trial was used for this study. The row-plot field trial comprised 18 perennial ryegrass breeding lines and reference cultivars with a random replication of each cultivar (from 7 to 10 replicates) contained a total of 160 plots and was established in four primary rows of 40 plots each (with 1 m inter-row spacing) in June 2017. Each plot had dimensions of 1.8 × 4 m with 48 plants from one cultivar and was made up of three rows (16 plants per row and 0.25 m spacing between plants). Rows were spaced at 0.6 m. The experimental data were collected at three time points (June, October, and December) in 2019, which corresponded to Winter, Late Spring, and Summer seasons, respectively, spanning most of the growing season of perennial ryegrass in Australia. Data collection date was determined when the plants were at the three leaf stages, which is the appropriate harvesting time in the Winter and Late Spring seasons. For the Summer dataset, data capture was during the reproductive development phase to examine biomass estimation performance from sensors under this stage. Data collection consisted of harvested biomass using a destructive method and phenotyping data from aerial and ground-based phenotyping platforms (Figure 1). All phenotyping data were collected a day before harvest day in the order of crewed ground vehicle, UAV, and UGV. The priority was given to the crewed ground vehicle because it was driven by a human operator and was conducted in the early morning to avoid sunlight and high temperatures, whereas the UGV was driven in the afternoon. The UAV was operated in the middle of the day when it has sunny and clear or cloudy conditions to minimize the influence of solar irradiance and shadows. For the October dataset, two of the four mowers had a technical issue caused by a cutting plate which caused inaccurate FM cuts from the plants in the first 80 plots, therefore these plots were removed from the dataset.

### Data collection and analysis

#### Normalized difference vegetative index: Uncrewed aerial system

For NDVI extraction, aerial multispectral images of the field trial were taken at nadir by a MicaSense RedEdge-M (MicaSense In., Seattle, WA, United States) camera mounted on a DJI Matrice 100 quadcopter (DJI Technology Co., Shenzhen, China). The flight missions were created on Pix4DCapture software (Pix4D SA, Switzerland) and enabled the DJI Matrice 100 to fly at 30 m flight attitude, a speed of 2.5 m/s (9 km/h), and with forward and sideways image overlap of 75%. The MicaSense RedEdge-M multispectral camera captured five spectral narrowband images (Blue at 465–485 nm, Green at 550–570 nm, Red at 663–673 nm, Red Edge at 712–722 nm, and Near-infrared at 820–860 nm) with a ground sample distance of 2.08 cm/pixel on fast mode according to the MicaSense capture settings of the DJI Matrice 100. For further image processing, a radiometric calibration tarp (Tetracam Inc., Chatsworth, CA, United States) with five reflectance percentages (3, 6, 11, 22, and 33%) and multiple ground control points (GCPs) within the trial (Supplementary Figure 1) were used to calibrate the reflectance values of the acquired images for all bands and perform the georectification. Image processing, georectification, and orthomosaics were conducted with the Pix4Dmapper 4.2.16 software (Pix4D SA, Switzerland). The output image of each band was exported in the coordinate reference system (CRS) WGS 84 54S and calibrated to reflectance values by linear regression equations from the calibration tarp. The NDVI values were calculated and generated in the QGIS 3.4.15 software (QGIS Geographic Information System, Open Source Geospatial Foundation Project)^1^ by the following equation:


(1) $$\mathrm{NDVI}=\frac{\mathrm{NIR}-\mathrm{RED}}{\mathrm{NIR}+\mathrm{RED}},$$

where NIR and RED are spectral bands at wavelengths 820–860 and 663–673 nm, respectively.

Polygons of individual rows were created based on the NDVI image and linked to identifiers (ID) following Gebremedhin et al. (2020). Polygons were then used to extract per row NDVI, plant height, and LiDAR data for downstream analysis.

#### Sonar plant height: Crewed ground vehicle

Our trial configuration was similar to Gebremedhin et al.’s (2019a) and plant height of rows was measured with ultrasonic sonar Baumer UNDK 30 U6103/S14 sensors (Baumer Group, Frauenfeld, Switzerland) mounted on a 1.45 m wide tailored steel frame that adapts to the front of a crewed ground vehicle (PhenoRover) and extracted by following their method. In short, the PhenoRover is a Polaris Ranger 6 × 6 side-by-side vehicle (Polaris Industries Inc., Medina, MN, United States) that was equipped with a custom-developed height acquisition system consisting of six Baumer UNDK 30 U6103/S14 sonar sensors, a Trimble Real-time Kinematics—Global Navigation Satellite System (RTK-GNSS), and a Campbell Scientific CR3000 datalogger (Campbell Scientific, Inc., Logan, UT, United States). Six sonar sensors were mounted in a metal front chassis for three-row measurements where two are in one row and at 0.6 m apart of each other as same as to the ground. All six sensors were set at 10 Hz for fast measurement, and thereby, the driving speed of vehicle was aimed to around 1.4 m/s (5.04 km/h) to ensure that the height measurements can be taken every 5–10 cm.

#### Lidar data metrics: Uncrewed ground vehicle

The DairyBioBot is a UGV developed at Agriculture Victoria Research, Hamilton SmartFarm, Victoria, Australia, which was equipped with the LMS400 2D LiDAR (SICK Vertriebs-GmbH, Germany) to capture plant structure in the field (Nguyen et al., 2021). In each planned mission, the DairyBioBot was driven following the specific path capturing two rows at a time in our experimental field trial at a speed of 1.2 m/s (4.3 km/h) while recording coordinates with its Real-Time Kinematic Global Position System (RTK GPS) at 5 Hz. At 1.127 m height above the ground cover, the LiDAR sensor was set to scan an angular range from 55° to 123° with a scan resolution of 1° to have a field of view (FOV) of two rows per scan. To ensure high plant resolution, the scan frequency was set at 500 Hz to accurately measure plant volume. The processing of LiDAR plant volume (LV) per row/plot was described in Nguyen et al. (2021). Briefly, raw LiDAR data and GPS coordinates were captured at two different frequencies and were processed and merged based on their time stamps. This generated a data point cloud that was visualized on the aerial image of the field trial and plant volume was calculated per polygon (per row/plot). LiDAR point density (LV_Den) was investigated as a potential plant density parameter, which is defined as a ratio of the number of the acquired data points (with cutoff threshold of 5 cm from the ground) over the total points for scans per row/plot. Further, LiDAR plant height (LV_PH) per row and plot was calculated as the average height measurement within a row polygon and three rows in a plot.

#### Biomass data: Destructive mechanical harvest

The ground-truth fresh biomass data were collected using a destructive mechanical harvest cut at 5 cm above the ground. To estimate biomass at the row and plot level, each individual row was manually cut by a stranded lawn mower (Model: HRX217K5HYUA; Engine capacity—190 cc; Honda Motor Co., Ltd., Tokyo, Japan) and bagged in a plastic bag labeled with a Row Identity (RowID) and Plot Identity (PlotID). After harvest, all 480 FM samples were weighed using a Mettler Toledo GmbH scale (Model: ICS6x5–1; Mettler-Toledo Ltd., Toledo, OH, United States). Biomass data per plot were calculated as the sum of FM of three rows in a plot based on their PlotID. In the Winter (June) dataset, FM was only measured at the whole plot level.

### Predictive modeling

**Figure 2:**
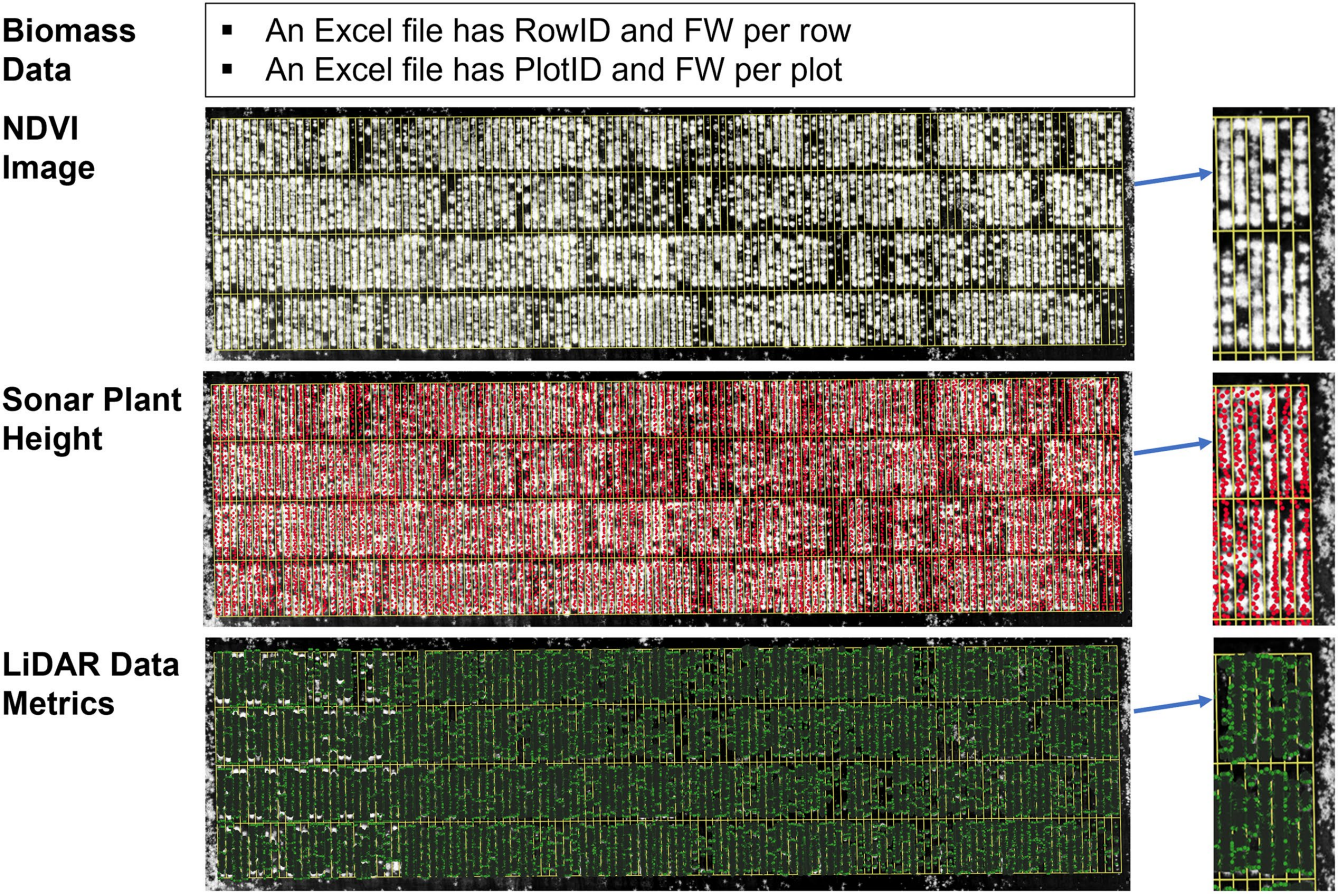
Each processed dataset consists of biomass data, normalized difference vegetation index (NDVI) image, Sonar plant height, and LiDAR data metrics (plant height, volume, and density) for each time point. In biomass data, the identity of each row and plot is classified in the RowID and PlotID columns versus its corresponding fresh mass (FM) value.

All digital plant phenotypes extracted from sensors (Figure 2), including NDVI, Sonar_PH, LV, LV_Den, and LV_PH, were initially tested by estimating linear regressions with FM biomass in within and combined season datasets. Using *PerformanceAnalytics* package in R version 4.0.3 (R Core Team 2020, Vienna), each variable’s ability to estimate biomass was evaluated with the Pearson’s correlation coefficient (*R*). For biomass estimation in ryegrass species, multiple linear regression (MLR) is an appropriate method to make predictive modeling (Martin et al., 2005; Giri et al., 2019). The MLR equation can be represented by the following formula:


(2) $$y=b_{0}+b_{1}x_{1}+b_{2}x_{2}+\cdots +b_{n}x_{n}+\varepsilon$$

where: *y* is the measured fresh biomass (*g*),

$b_{0},b_{1},b_{2},and\;b_{n}\;$ are parameters of the model,

$x_{1},x_{2},and\;x_{n}\;$ are an exploratory variable,

ε is the error of prediction, and n is the number of variables.

All MLR models combined (without interactions) from our phenomic variables were established using the best subsets selection method using the *leaps* R package. This method computes all models using a specified subset of predictors and presents the best-fitting model within each set. The adjusted coefficient of determination (Adjusted *R*^2^), which is the proportion of variation in predictor values, was used to first assess all the best-fitting models in each dataset. For further evaluation, the summer dataset is a good test set as it represents a wide variation in the plant fraction between vegetative and reproductive parts of 480 rows (160 plots), which therefore can be used to identify different best models of different subsets based on the statistical criteria for multiple variables, including Adjusted *R*^2^, Mallows’s Cp criterion (Cp), and Bayesian information (BIC). The best subset was selected by higher Adjusted *R*^2^, lower Cp, and BIC values along with a smaller number of variables. According to physics, the mass of an object can be calculated by multiplying volume by density. Volume can predict biomass accurately (e.g., an LV predictor), but the accuracy of biomass estimation in grass species can be affected by inconsistent plant density, which can be caused by many factors, such as leaf area, tiller density, leaf mass as well as chlorophyll content (Van Loo, 1993; Matthew et al., 1996; Sbrissia et al., 2001). Plant density is an unknown variable in biomass prediction, but could be partly explained by LV_Den, NDVI, or the combination of LV_Den and NDVI variables. We developed models (M3 to M6) to determine the best biomass prediction model based on these hypotheses (Table 1). Additionally, the published model at the proximal individual plant level by Gebremedhin et al. (2019a) was used to compare LiDAR+NDVI ( FM~LV_PH×NDVI ) performance versus LiDAR only models (FM~LV_PH ). The best performing model was selected based on the statistical metrics including Adjusted *R*^2^, Residual Standard Error (RSE), and *F*-statistic (*F*), but the best model was also considered with our specified optimal criteria such as the number of variables and number of sensor technologies in a prediction equation, and the consistency of variables in different datasets. To test the prediction accuracy and robustness of the selected best model, multiple cross-validations approaches using *caret* R package with different settings from repeated *k*-fold with ten times, leave-one-out to random split was used to split the dataset into training and testing sets. Significant statistical parameters including root mean squared error (RMSE), the determination of coefficient (*R*^2^), and mean absolute error (MAE) were presented to assess the overall prediction performance of the selected model.

**Table 1:** Multiple linear regression models used to compare the predicted and measured fresh weight (FM) samples.

Model	Equation
M1	FM~NDVI+Sonar_PH+NDVI×Sonar_PH
M2	FM~NDVI+LV_PH+NDVI×LV_PH
M3	FM~LV×NDVI
M4	FM~LV×LV_Den
M5	FM~LV×(LV_Den+NDVI)
M6	FM~LV×(LV_Den×NDVI)

Predictor variables include normalized difference vegetation index (NDVI), average plant height (Sonar_PH), LiDAR plant height (LV_PH), LiDAR plant volume (LV), and LiDAR point density (LV_Den).

### Statistical tools

All plots and statistical analyses were performed in the R environment through R version 4.0.3 (R Core Team 2020, Vienna). Besides the base and dependencies packages, necessary packages for data analysis were used *ggplot2* for plotting, *PerformanceAnalytics* for advanced plotting with Pearson’s correlation coefficient in one figure, *tidyverse* for data manipulation and visualization, *caret* for computing cross-validation methods, and *leaps* for the best subset selection.

## Results

### Relationship between sensor-based phenotypes and biomass

**Figure 3:**
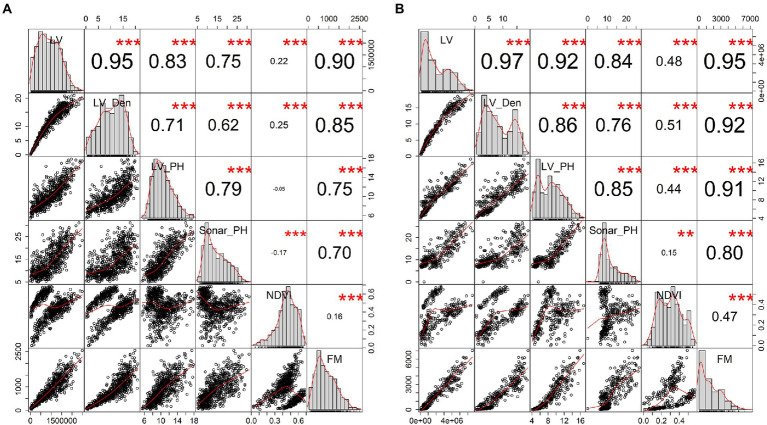
Evaluation of the relationships between plant digital parameters extracted from sensor-based phenomics and harvested fresh biomass (FM) in the combined seasonal dataset. Panel **(A)** presents 720 samples at row level and panel **(B)** for 368 samples at the plot level. In the panel, histogram windows display the distribution of individual parameters on the diagonal of the panel, whereas the bivariate scatter plots with a red fitted line and Pearson’s correlation coefficient with the significant level as stars at the top and bottom of the diagonal, respectively. Star symbols are “^***^,” “^**^” and “^*^” for *p-values* are ≤ 0.001, 0.01, and 1.0. LV, LiDAR plant volume; LV_Den, LiDAR point density; LV_PH, LiDAR plant height; Sonar_PH, ultrasonic plant height; NDVI, multispectral normalized different index; and FM, manual harvested fresh biomass.

Sensor-based phenotype distributions and correlations with destructive FM were visualized for the combined dataset across all three seasons (Winter, Late Spring, and Summer, Figure 3). All digital parameters were significantly correlated with FM (*R* > 0.7, *p* ≤ 0.001), except NDVI from multispectral images, which was poorly correlated at 0.16 and 0.47 for the row and plot levels, respectively. Based on correlations with FM, biomass predictors could be ranked from LV, LV_Den, LV_PH, Sonar_PH to NDVI. Both LV and LV_Den extracted from the LiDAR sensor were highly correlated with fresh FM at both levels with significant correlations of 0.90–0.95 and 0.85–0.92, respectively. Similarly, in the seasonal datasets, the results showed that the LiDAR-derived metrics such as LV and LV_Den were most correlated with FM (*R* = 0.76–0.97, Table 2) and followed by NDVI. In addition, LV and LV_Den had a strong correlation at both the row and plot levels with a minimum *R* = 0.95, indicating that they capture similar information. Sonar_PH in Late Spring was poorly correlated with FM and was much more variable across seasons than other parameters. These findings suggest that a model derived from the LV and LV_Den parameters could be suitable for predicting FM, but incorporating with NDVI could potentially make predictions more robust across the seasons.

**Table 2:** A summary table of Pearson’s correlation coefficient (R) for the empirical relationship between each plant digital parameter and harvested fresh biomass in the seasonal dataset at the row and plot level.

Row level		Plot level		
Parameters
	Late spring	Summer	Winter	Late spring	Summer
LV	0.94	0.84	0.90	0.97	0.86
LV_Den	0.92	0.76	0.83	0.95	0.77
LV_PH	0.64	0.61	0.84	0.80	0.69
Sonar_PH	0.11	0.52	0.61	0.01	0.55
NDVI	0.85	0.80	0.75	0.87	0.81

### Best subset modeling

**Figure 4:**
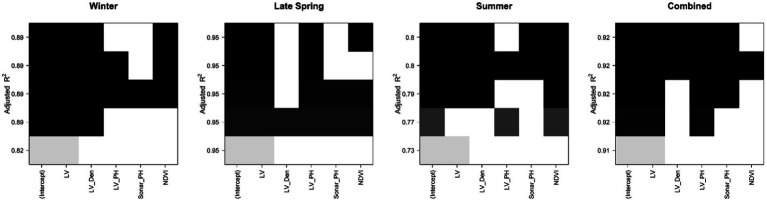
The color map of Adjusted *R*^2^ values comparing the performance of models involving five senor-based predictors (from 1 to 5 predictors and an intercept variable per subset) and fresh biomass in each dataset at the plot level. Each predictor is presented by a colored square and arranged with other predictors of the same color. Color darkness is proportional to adjusted *R*^2^ values, where black is highest.

We tested all models involving our five sensor-based parameters to find a parsimonious combination with good performance and the fewest parameters and sensor types. In general, there were usually several models with very similar performance (*R*^2^, Figure 4). Except for the Summer season, LiDAR-derived parameters achieved the same accuracy as models including NDVI. Sonar_PH was least included in top models, indicating its limited utility. For vegetative stage FM, the high prediction was estimated by the combination of the LV and LV_Den in Winter but in Late Spring, LV on its own achieved up to 0.95 Adjusted *R*^2^. In Summer, at the reproductive stage, *R*^2^ varied substantially from 0.73 to 0.8 with different subsets of parameters. Models with all parameters achieved *R*^2^ of 0.80, while a model excluding Sonar_PH resulted in a comparable *R*^2^ of 0.79. Using only LiDAR parameters reduced *R*^2^ to 0.73 in Summer, indicating some loss of information. These *R*^2^ trends were confirmed with other model performance metrics Mallow Cp and BIC (Supplementary Figure 2). Similar results were also shown at the row level in Supplementary Figures 3, 4. In the combined seasonal dataset, the standalone LV variable provided an excellent prediction at the Adjusted *R*^2^ of 0.91 with little improvement when combined with the other parameters. Adding sensor parameters other than LiDAR did not improve *R*^2^.

### Comparisons of six models including parameter combinations

**Figure 5:**
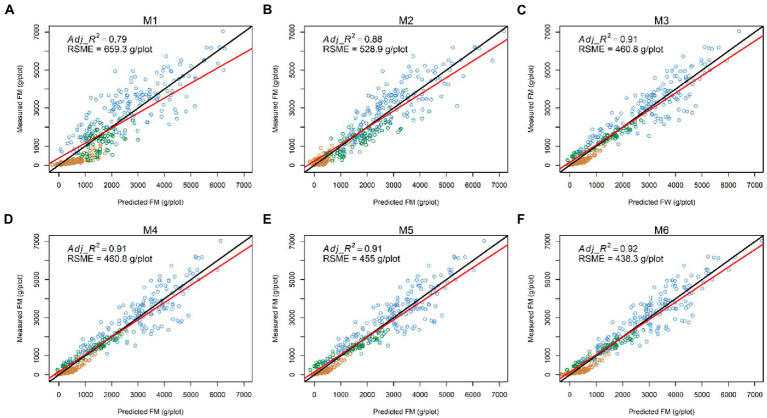
Comparisons between the measured and predicted fresh biomass (FM) at the plot level (*N* = 386) in the combined dataset from six prediction models: **(A)** M1: FM∼NDVI+Sonar_PH+NDVI×Sonar_PH, **(B)** M2: FM∼NDVI+LV_PH+NDVI×LV_PH, **(C)** M3: FM~LV×NDVI, **(D)** M4: FM~LV×LV_Den, **(E)** M5: FM~LV×(LV_Den+NDVI), **(F)** M6: FM~LV×(LV_Den×NDVI). The red and black lines represent the best-fit linear regression and 1:1 line, respectively. Adj_*R*^2^: adjusted coefficient of determination; RSME: root mean square error.

**Figure 6:**
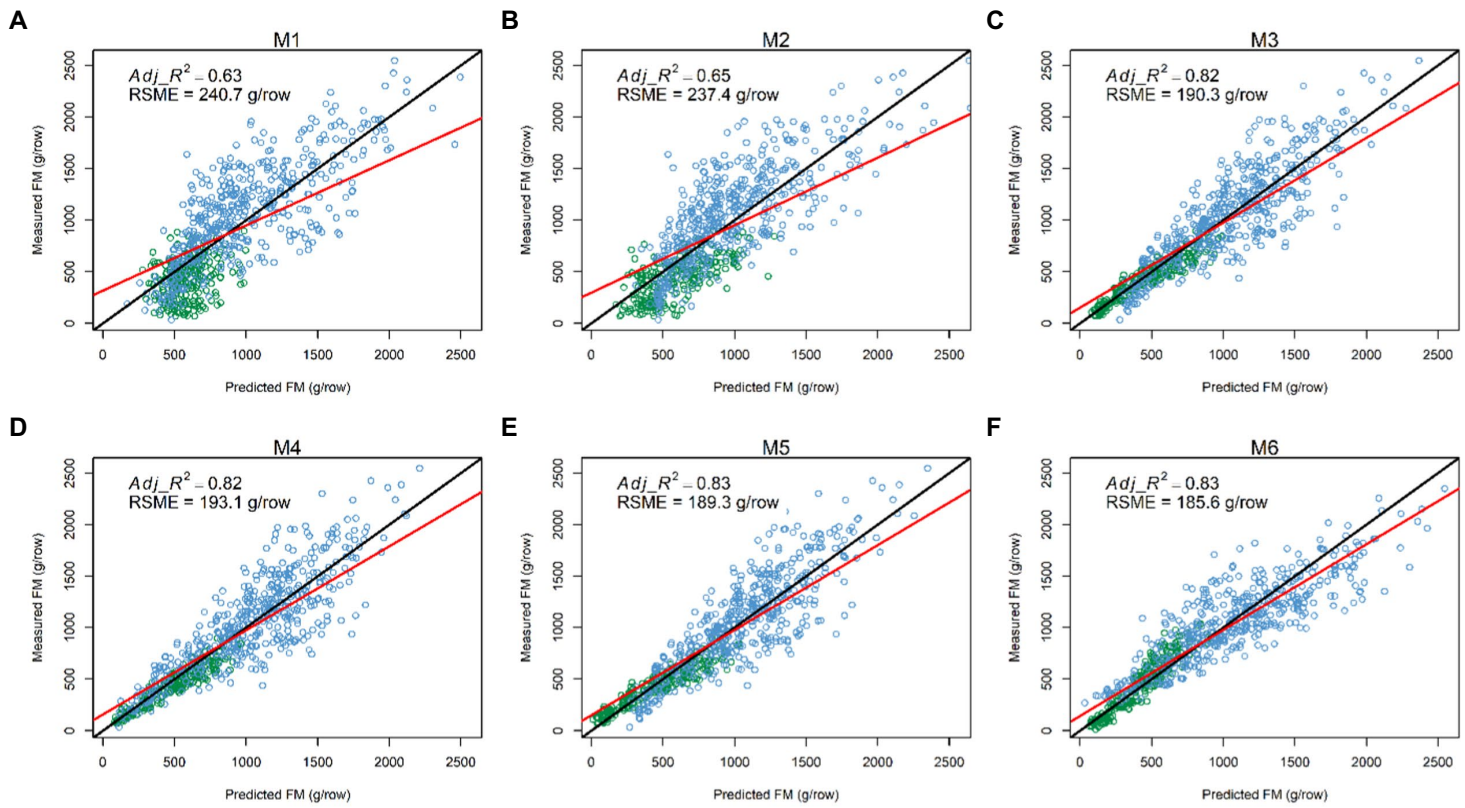
Comparisons between measured and predicted fresh biomass (FM) at the row level (N = 720) in the combined dataset from six models: **(A)** M1: FM~NDVI+Sonar_PH+NDVI×Sonar_PH, **(B)** M2: FM~NDVI+LV_PH+NDVI×LV_PH, **(C)** M3: FM~LV×NDVI, **(D)** M4: FM~LV×LV_Den, **(E)** M5: FM~LV×(LV_Den+NDVI), and **(F)** M6: FM~LV×(LV_Den×NDVI). The red and black lines represent the best-fit linear regression and 1:1 line, respectively. Adj_*R*2: adjusted coefficient of determination; RSME: root mean square error.

Having explored which parameters were useful in 3.2, we wanted to investigate whether including combinations (sum or products) of parameters could further improve model fit. All models incorporating an LV predictor had a clear improvement of R^2^ over models with only NDVI and Sonar_PH/LV_PH (M1 and M2, respectively) at both the row and plot level in the combined seasonal dataset (Figures 5, 6; Table 3). The observed *R*^2^ from M3 to M6 was at least 0.82 and 0.91 for the row and plot level, respectively, where model M1 and M2 estimated 0.63–0.65 for the row level and 0.79–0.88 for the plot level. Model M2, including LV_PH, performed slightly better than model M1, including Sonar_PH, across all datasets. Among all prediction models, the best performing model was model M6: FM~LV×(LV_Den×NDVI) on both assessment levels, but more simple models with two variables (e.g., model M3: FM~LV×NDVI and M4: FM~LV×LV_Den ) were similar in *R*^2^, except in Summer. This indicates that models M3 and M4 may be competitive simpler prediction options.

**Table 3:** Comparison of regression performance of models to predict biomass at row and plot levels in seasonal datasets.

Row level				Plot level					
Model	Late spring		Summer		Winter		Late spring		Summer	
	Adj_*R*^2^	RMSE	Adj_*R*^2^	RMSE	Adj_*R*^2^	RMSE	Adj_*R*^2^	RMSE	Adj_*R*^2^	RMSE
M1	0.73	86.17	0.69	216.5	0.73	99.90	0.75	233.3	0.75	563.0
M2	0.80	78.38	0.72	210.3	0.86	79.22	0.88	175.6	0.79	527.0
M3	0.89	60.79	0.74	205.0	0.88	73.34	0.95	118.0	0.78	538.2
M4	0.89	60.44	0.71	212.2	0.91	65.89	0.95	117.8	0.74	572.7
M5	0.90	59.66	0.76	200.9	0.91	64.61	0.95	115.6	0.81	505.8
M6	0.90	59.51	0.76	200.3	0.92	62.33	0.95	115.0	0.81	504.4

Adj_*R*^2^: adjusted coefficient of determination. RMSE: root mean square error. All models significant *p*-values ≤ 0.001.

### Detailed evaluation and validation of parsimonious models

To select the best prediction model, model M3: FM~LV×NDVI and M4: FM~LV×LV_Den were evaluated for their overall prediction performance (Table 4). Similar performance was observed from both models at row and plot levels for all metrics. Especially, in the Summer dataset, the plants were at the reproductive stage and biomass included flowering heads. Model M3 resulted in a slightly higher *R*^2^ and was lower for the other parameters (e.g., RSE, AIC, BIC, and PER) than model M4 (Table 4). Since NDVI measures the green vegetative density of plants, model M4 produced a better prediction of biomass including green material and flowering heads than model M3 incorporated with LV_Den (measured point density of plants). However, NDVI is extracted from a multispectral camera integrated on an aerial platform, whereas the LV and LV_Den were from the LiDAR sensor on the same ground platform. Considering that M3 and M4 perform very similarly in other seasons, the increase in *R*^2^ from the capture of NDVI in Summer may not be worthwhile economically and M4 may be the best pragmatic model.

**Table 4:** Detailed comparison of Model M3: FM~LV×NDVI and M4: FM~LV×LV_Den at the plot level.

Row level						Plot level							
Statistic parameter	Late spring		Summer		Combined		Winter		Late spring		Summer		Combined	
	M3	M4	M3	M4	M3	M4	M3	M4	M3	M4	M3	M4	M3	M4
Adjusted *R*^2^	0.89	0.89	**0.74**	**0.71**	0.82	0.82	0.88	0.91	0.95	0.95	**0.78**	**0.74**	0.91	0.91
RSE	64.7	64.3	**239**	**252**	210	214	78.8	69.8	123	122	**613**	**672**	484	484
AIC	2,686	2,683	**6,622**	**6,675**	9,747	9,774	1,485	1,454	1,000	1,000	**2,512**	**2,541**	5,599	5,599
BIC	2,697	2,694	**6,634**	**6,687**	9,761	9,788	1,494	1,463	1,008	1,007	**2,521**	**2,551**	5,611	5,610
PER	0.15	0.15	**0.22**	**0.24**	0.24	0.25	0.35	0.31	0.09	0.09	**0.19**	**0.29**	0.28	0.28

The bold numbers represent the main difference between the two models. FM: harvested fresh biomass; LV: LiDAR plant volume; LV_Den: LiDAR point density; NDVI: normalized difference vegetation index; Adjusted *R*^2^: adjusted coefficient of determination; RSE: relative standard error; AIC: Akaike information criterion; BIC: Bayesian information criterion; and PER: prediction error rate.

We performed cross-validations to explore the predictive ability of Model M4 ( FM~LV×LV_Den) in the combined dataset. At the plot level, M4 showed strong *R*^2^ consistency from 0.91 to 0.92, with a small variation of RSME and MAE ranging from 456.41 to 507.31 g/plot and 319.11 to 359.93 g/plot across all CV tests (Supplementary Table 1). Similarly, on the row level, the estimated *R*^2^, RSM, and MAE were obtained from 0.80 to 0.83, 209.42 to 220.77 g/row, and 149.58 to 158.10 g/row, respectively. Based on assessing the statistical significance through all three CV approaches with different settings, M4 predicted biomass well.

## Discussion

We developed and validated within and combined season prediction models, based on sensor data, to assess across-season biomass yield of perennial ryegrass. Under different growth conditions, growth responses measured as biomass production over time vary considerably. Particularly, climate metrics such as rainfall and temperature change across seasons and influence biomass accumulation in seasonal patterns. In addition, perennial ryegrass has a complex plant structure and non-uniform growth habit from individual to the group (e.g., row or sward) through seasons and years. These changes can influence the effectiveness of each sensor-based technology differently. However, the selection of cultivars with a high yield trait is highly dependent on the accurate biomass estimation across repeated measurements to evaluate robust yield performance. Our study explored the predictability of digital structural and vegetative predictors at different scales of biomass assessment across a within and combined season dataset to understand forage biomass estimation in response to the season changes. Our aim was to achieve an accurate and robust across-season prediction model.

Our results demonstrated that LV and LV_Den were the strongest predictors of forage biomass. A consistent strong relationship with biomass was observed across all seasonal and combined-seasonal datasets at both row and plot levels. Each digital phenotype still has its own drawbacks. While LV and LV_Den were consistently amongst the best phenotypes for biomass prediction, the rank of the other phenotypes widely varied from specific to multiple growing seasons. NDVI performed well within and over seasons but recorded a dramatic drop in the combined-season dataset. Physical parameters with a larger measurement range performed more robustly than vegetation indices. Although the sample population was large and chosen to represent the diversity of growth and development in perennial ryegrass, this diversity is often related to relatively small ranges in spectral diversity. This phenomenon is also seen when relating chemical diversity to spectral characteristics in ryegrass. The small numerical NDVI ranges are a limitation to explain biomass changes on its own across growth stages and/or seasons. Better observations from sensor predictors in the vegetative stages (such as in Winter and Late Spring) were obtained than in the reproductive stage (Summer). Therefore, identification of major predictive variables is crucial to get a robust across-season model at multiple temporal points and scales, also taking into account a desire to minimize the number of parameters and required sensor-based platforms.

Traditionally, modeling by combining all the best performing datatypes can achieve the maximum predictive ability by compensating each other’s drawbacks, but may not reflect the true plant responses over seasons for biomass. From the color maps representing the best subset selection (Figure 4), the best combined models within a single/combined season can easily be determined with each of the datasets, this optimization was not always apparent in reviewing adjusted R^2^. This usually occurred if there were many similar performing predictive parameters in a set (like vegetative indices or VIs); but in this study, they were completely different from each other and in different relationships to biomass. Consequently, it will not guarantee that the best-fit model predicts well in one season and will also perform well in another season and future dataset. Therefore, developing prediction models based on the combining method would not necessarily produce a robust estimation that can apply across seasons. Since LV, LV_Den, and NDVI were the best predictors and have a strong empirical relationship with each other, they come closer to interpreting mass theory, and also proved as a good combination in the deeper model evaluation by the best subset selection method in the summer dataset. Our proposed models have demonstrated that the estimations can be similar as combined models without the effort of using all acquired parameters. The best performing prediction model was M6: FM~LV∗LV_Den∗NDVI which provided an excellent correlation with harvested biomass within and across seasons. However, although M3 and M4 differed from M6 by missing some interactions, they produced a similar prediction performance. The small increase when including NDVI could be due to NDVI capturing color differences of flowering heads versus leaves in the vegetation in the summer season (reproductive stage). Measurement in the summer is usually to score the heading date of the reproductive stage to select the late reproductive varieties. However, most breeding programs would cut before heading date for yield observation. The R^2^ improvement from including NDVI may not warrant the cost expense in investing in an aerial platform to capture NDVI. In terms of practical application, model M4: FM~LV×LV_Den then is more likely the best prediction model, as it requires only one data source. The reliability of model M4 was validated through different cross-validation and proven by the strong consistency of *R*^2^ at around 0.92, with a small variation on RSME and MAE ranging from 456.41 to 507.31 g/plot and 319.11 to 359.93 g/row. Hence, our model M4 can be used as an across-season prediction model for measuring biomass in perennial ryegrass.

Most published models were developed on the plot or sward level, in which sub-setting the samples is commonly used to scale up the assessment level. To date, only one study in the spaced plants was published using the prediction model (M1) of 480 plants over 2 years and then scaling its validation to the plot level (Gebremedhin et al., 2019a). Similar to that trial configuration, our best performing M6 model had a significantly higher accuracy at the row (Adj_*R*^2^ = 0.70–0.90, RSME = 59.61–200.3 g/row) and plot levels (Adj_*R*^2^ = 0.81–0.95, RSME = 62.33–504.4 g/plot) compared with M1 in the previous study. The M1 performance in this study was similar as reported previously. We can expect that future performance of the M1 model would be similar having now observed it in similar trials across multiple years. Larger RSME was observed when biomass was harvested as the whole row, instead of the sum of harvested single plants in a row. For the plot level, the estimated correlations from our proposed models were slightly higher than the previous studies, for example, *R*^2^ = 0.78 from LV_PH × NDVI in tall fescue (Schaefer and Lamb, 2016), *R*^2^ > 0.8 from all Crop Volume (CV) × VIs models in wheat (Banerjee et al., 2020), and *R*^2^ = 0.90 of the Vegetation Index Weighted Canopy Volume Model in soybean (Maimaitijiang et al., 2019). Since plant height is easy to measure and extract from inexpensive sensors/cameras, the current trend of developing prediction models is based on the combination of plant height with other parameters (like VIs). While the VIs remain the fixed limitation, the main improvement of model accuracy is therefore depended on the plant height capacity (Togeiro de Alckmin et al., 2021). Many studies showed that higher accuracy of plant height was measured by being closer to the ground level than aerial cameras (Madec et al., 2017; Wang et al., 2018; Yuan et al., 2018a). Ground-based LiDAR data can provide more detailed information of complex height profiles than a single data point from the popular low-cost ultrasonic Sonar sensors. As a result, LV_PH simply dominated Sonar_PH in this study as well as the others (Yuan et al., 2018a; Thompson et al., 2019; Walter et al., 2019). Noticeably, the poor-to-moderate performance of plant height started from the row to plot level as the decreasing diversity of height between plants. Therefore, the height-based models are more suitable to apply at swards/paddocks but may not be a good approach in the single plants/rows. Our developed models based on ground-based LiDAR data have demonstrated their better prediction ability at the row and plot level, possibly the single plants level, which indicated the broader application of those models can be used at the different scales from plants to plots in the breeding trials.

To improve the prediction accuracy of the developed models, it is advisable to further study the water (moisture) content as it also contributes to the overall weight of FM. This may explain the lower summer correlations compared to other seasons, which agrees with rainfall as the main discriminating factor for reducing prediction error for tropic perennial grasses (Youkhana et al., 2017). The proposed model M4 still has the disadvantage of predictive speed due to the longer data collection time of UGV compared to the aerial platforms. Current settings allow the UGV to move between two rows at a time at a speed 1.2 m/s (4.3 km/h) to capture high-resolution plant data. Accurate and reliable biomass measurement takes precedence over data collection time in the cultivar selection process, especially applying the genomic selection method. New studies have begun to integrate a compact LiDAR sensor with RGB/multispectral camera (or newly as multispectral LiDAR camera) on an aerial platform. The advent of this technology will provide an all-in-one platform that rapidly measures not only biomass but additional important traits (e.g., nutrition, quality, and persistence; Roitsch et al., 2019). However, this technology is mainly designed for environmental, topographical, and mining surveying but not for precise agriculture yet (Li et al., 2021). The wide scan area and measurement calibration are still major limitations in measuring LV for forage species with aerial LiDAR technologies (Chen et al., 2017; Yan et al., 2020). In future research, this study may form the opening of using multispectral LiDAR data for a comprehensive yield and nutritive value prediction model of the perennial ryegrass growth cycle in large field trials.

The application of sensor technology in high-throughput plant phenotyping to date has been mainly by using derived digital parameters for modeling and prediction traits of interest such as biomass, plant height, or nutritive values. It is essential to find the correlation relationships; therefore, model construction and cross-validation are still necessary where tradition way of data collection is inevitable. It would be important to understand the underlying biophysical and/or biochemical meanings of digital parameters. This may lead to direct digital phenotyping without converting them to traditional traits in the future. For example, LV and LV_Den can directly monitor plant growth and production in response to growing conditions.

## Conclusion

This study demonstrated the excellent ability of ground-based LiDAR data from UGV and compared it to other ground -and air-based sensor phenotyping platforms in estimating the across-season biomass yield of perennial ryegrass. LV and LV_Den extracted from LiDAR data strongly correlate with FM at a high level of accuracy that is robust within and combined seasons. In addition, LV is a key parameter in all best-fit subset models, and adding more predictive parameters resulted in only small improvements. Thus, the modeling approach based on the mass theory is more accurate than the traditional methods of predictive modeling in generating simple models with a small number of parameters. The developed models show a robust performance over seasons compared with the top best-fit subset models and models tested in other studies. In terms of practical application, the simple prediction model FM ~ LV × LV_Den is the best choice for a global prediction model of across-season biomass, as it requires data from only one sensor type. With a high precision of estimated biomass, it would assist the use of genomic selection in large populations to develop higher yielding cultivars.

## Data availability statement

The original contributions presented in the study are included in the article/Supplementary material; further inquiries can be directed to the corresponding author.

## Author contributions

PN, HD, KS, PB, JW, and GS conceived the research. KS, PB, PN, and JW designed and managed the field trial and resources of this study. HD supervised the study. PN analyzed all the data and drafted the manuscript. FS provided the automated polygons for rows and plots. HD and FS advised the formal analysis. PN and HD wrote the manuscript. KS, PB, JW, and GS reviewed and edited the manuscript. All authors contributed to the article and approved the submitted version.

## Funding

This study was funded by DairyBio, a joint venture of Agriculture Victoria, Dairy Australia, and the Gardiner Dairy Foundation. PN received a La Trobe University Scholarship.

## Conflict of interest

The authors declare that the research was conducted in the absence of any commercial or financial relationships that could be construed as a potential conflict of interest.

## Publisher’s note

All claims expressed in this article are solely those of the authors and do not necessarily represent those of their affiliated organizations, or those of the publisher, the editors and the reviewers. Any product that may be evaluated in this article, or claim that may be made by its manufacturer, is not guaranteed or endorsed by the publisher.

## Footnotes

^1^ http://qgis.osgeo.org

## Supplementary material

The Supplementary material for this article can be found online at: https://www.frontiersin.org/articles/10.3389/fpls. 2022.950720/full#supplementary-material
