# Supplementary material for: Within and combined season prediction models for perennial ryegrass biomass yield using ground- and air-based sensor data
Source: Front Plant Sci. 2022 Aug 8;13:950720. doi: 10.3389/fpls.2022.950720 (PMC9393552; doi:10.3389/fpls.2022.950720)
Supplement: Supplementary file 1 [file Table_1.DOCX]

Within and Combined Season Prediction Model for Perennial Ryegrass Biomass Yield using Ground- and Air-Based Sensor Data

Phat T. Nguyen^1,2^, Fan Shi^2^, Junping Wang^3^, Pieter E. Badenhorst^3^, German C. Spangenberg^1,2^, Kevin F. Smith^3,4^, and Hans D. Daetwyler^1,2^

^1^ School of Applied System Biology, La Trobe University, Bundoora, VIC, Australia

^2^ Agriculture Victoria, AgriBio, Centre for AgriBioscience, Bundoora, VIC, Australia

^3^ Agriculture Victoria, Hamilton Centre, Hamilton, VIC, Australia

^4^ Faculty of Veterinary and Agricultural Sciences, School of Agriculture and Food, The University of Melbourne, VIC, Australia

*** Correspondence: phat.nguyen@agriculture.vic.gov.au**

Keywords: perennial ryegrass, cross-season yield, high-throughput phenotyping, sensor, prediction model, unmanned vehicle, uncrewed

# Supplementary Figures


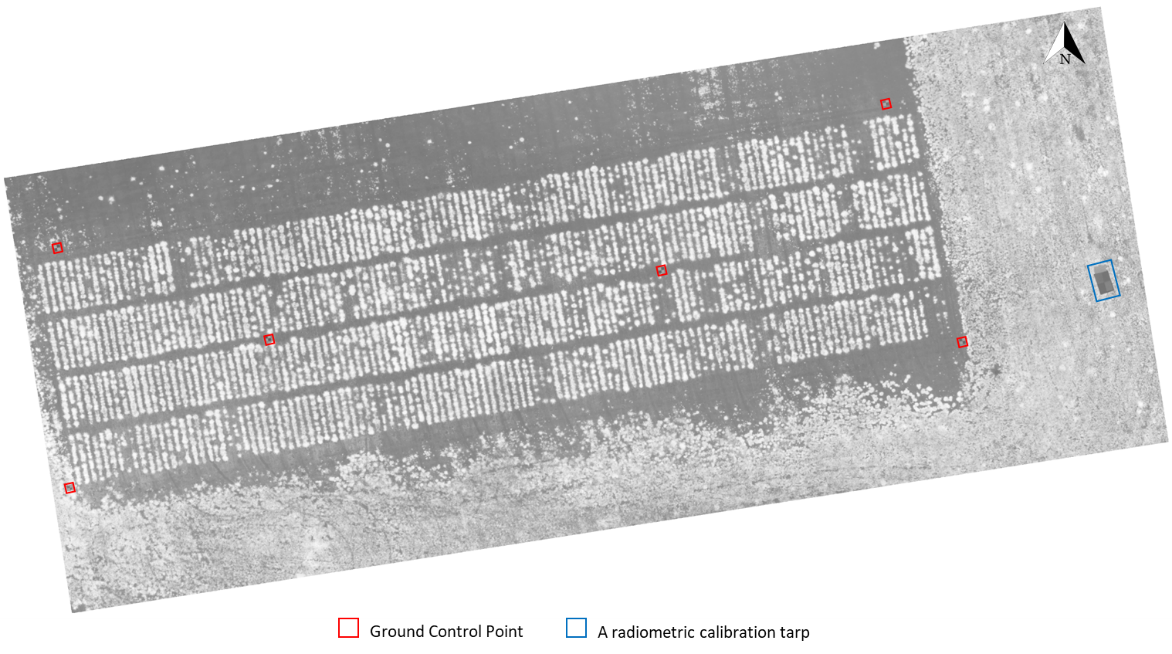


**Supplementary Figure 1.** Field preparation for Uncrewed Aerial Vehicle (UAV) image processing in the extraction of Normalized Difference Vegetation Index (NDVI) values.

**
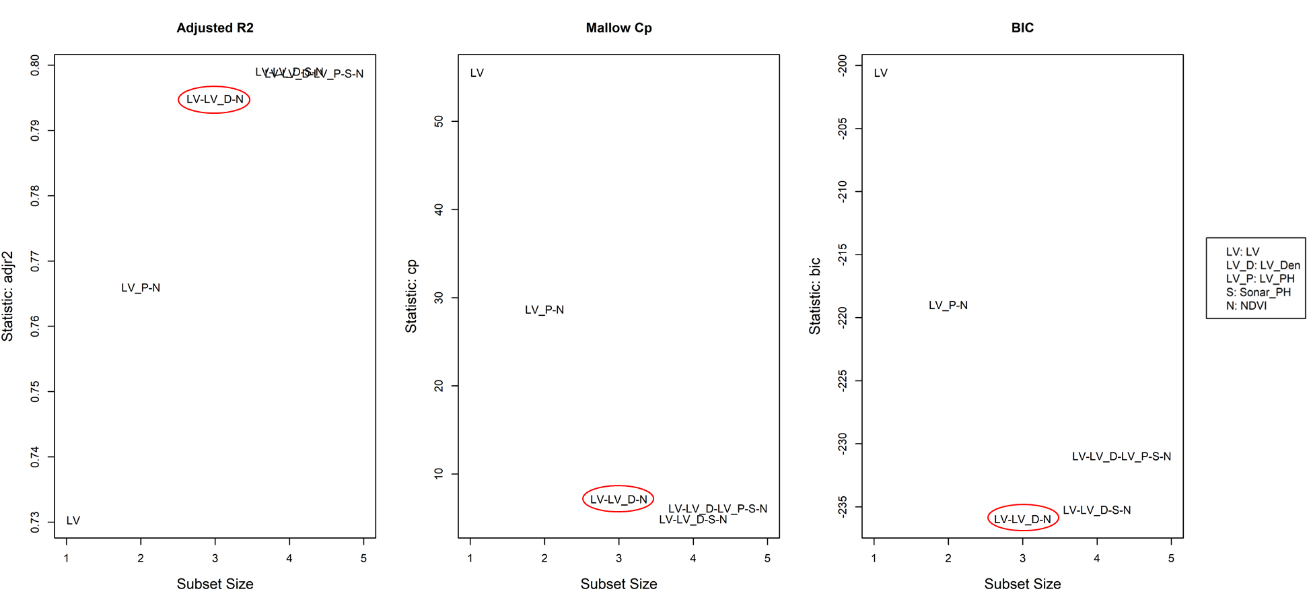
**

**Supplementary Figure 2**. Statistical analysis of the best subsets based on the subset size in the Summer dataset at the plot level. A red circle is used to highlight the selected best optimal subset based on good estimations on significant statistic parameters and fewer predictors in the subset in comparison with other high- performance subsets.

**
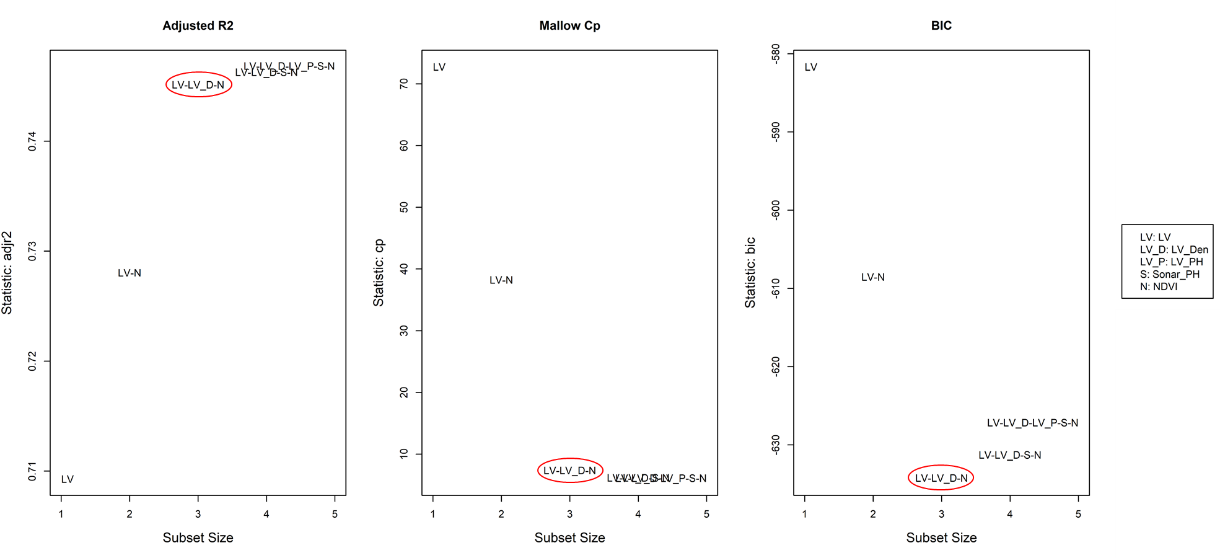
**
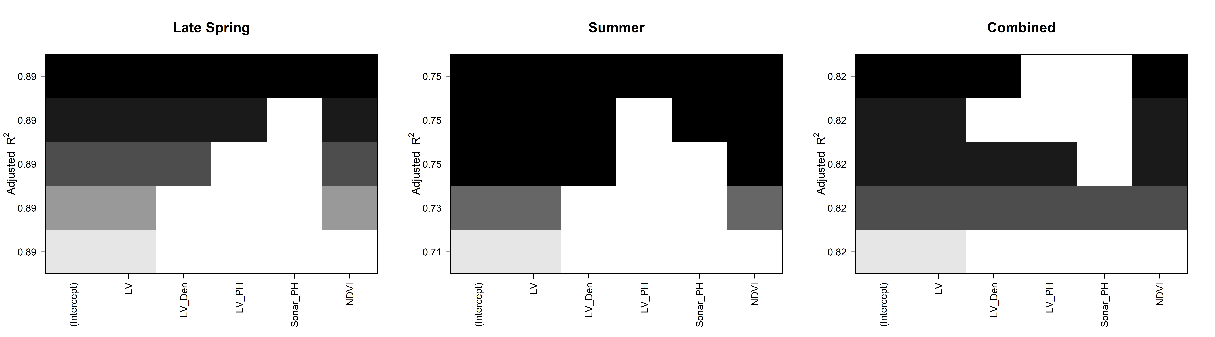
**Supplementary Figure 3.** The color map of Adjusted R^2^ values compares performance between best subsets for each number of predictors (from 1 to 5 predictors and an intercept variable per subset) and fresh biomass in each dataset at the row level. Each predictor is presented by a colored square and arranged with other predictors of the same color. The darkness of color is proportional to the adjusted R^2^ values, from black to light grey being highest to moderate.

**Supplementary Figure 4.** Statistical analysis of the best subsets based on the subset size in the Summer dataset at the row level. A red circle is used to highlight the selected best optimal subset based on good estimations on significant statistic parameters and fewer predictors in subset in comparison with other high- performance subsets.

**Supplementary Table 1.** The summary results of cross-validation (CV) approaches on the model M4 - $FM \sim LV\times LV\_Den$ including repeated k-fold, leave one out (LOOVC) and random split. The number of k-fold was set based on the number of samples on each level, 720 and 368 samples for row and plot level, respectively. In LOOVC tests, cultivars were randomly selected in 18 experimental cultivars sown in the field trials (e.g. named as FPH-001, FPH-007, FPH-011 and FPH-018) to take out for testing. Significant statistic parameters for assessing the model are Root Mean Square Error (RSME) in gram, coefficient of determination (R^2^), Mean Absolute Error (MAE) and Prediction Error Rate (PER).

|  |  | **Row Level** | | | | **Plot Level** | | | |
| --- | --- | --- | --- | --- | --- | --- | --- | --- | --- |
| **CV method** | **Setting** | **RSME** | **R^2^** | **MAE** | **PER** | **RSME** | **R^2^** | **MAE** | **PER** |
| *Repeated k-fold with 10 times* | k = 30 | 210.9 | 0.82 | 152.9 | 24.5 |  |  |  |  |
|  | k = 24 | 212.0 | 0.82 | 152.9 | 24.7 |  |  |  |  |
|  | k = 20 | 212.5 | 0.82 | 153.0 | 24.7 | 474.7 | 0.92 | 337.8 | 27.0 |
|  | k = 16 | 213.0 | 0.82 | 153.0 | 24.8 | 481.6 | 0.91 | 338.5 | 27.4 |
|  | k = 10 | 213.7 | 0.82 | 153.0 | 24.9 | 485.1 | 0.91 | 338.5 | 27.6 |
|  | k = 5 | 214.5 | 0.82 | 153.1 | 25.0 | 488.3 | 0.91 | 339.5 | 27.8 |
|  | k = 3 | 215.1 | 0.82 | 153.3 | 25.0 | 491.0 | 0.91 | 339.3 | 27.9 |
| *LOOVC* | 1 sample out | 215.1 | 0.81 | 153.0 | 25.0 | 490.7 | 0.91 | 338.4 | 27.9 |
|  | FPH-001 out | 220.8 | 0.80 | 158.1 | 24.8 | 497.6 | 0.91 | 339.1 | 27.3 |
|  | FPH-007 out | 216.7 | 0.82 | 154.0 | 25.5 | 494.9 | 0.91 | 341.1 | 28.7 |
|  | FPH-011 out | 218.8 | 0.81 | 155.4 | 25.4 | 497.4 | 0.91 | 341.8 | 28.3 |
|  | FPH-018 out | 210.0 | 0.81 | 149.6 | 24.9 | 477.5 | 0.91 | 330.0. | 27.7 |
| *Random Split* | 30% / 70% | 215.7 | 0.82 | 150.6 | 24.9 | 488.4 | 0.91 | 339.7 | 27.9 |
|  | 50% / 50% | 213.8 | 0.80 | 150.3 | 25.0 | 507.3 | 0.91 | 360.0 | 27.7 |
|  | 60% / 40% | 214.2 | 0.82 | 152.0 | 25.0 | 456.4 | 0.92 | 340.9 | 25.4 |
|  | 80% / 20% | 209.4 | 0.83 | 151.5 | 24.4 | 470.4 | 0.91 | 319.1 | 24.9 |
